# RELATIONSHIP OF SOCIAL SUPPORT AND SOCIAL ENGAGEMENT WITH COGNITION IN AN ETHNICALLY DIVERSE OLDER POPULATION

**DOI:** 10.1093/geroni/igad104.0570

**Published:** 2023-12-21

**Authors:** Ruth Tappen, Rebecca Koszalinski, David Newman

**Affiliations:** Florida Atlantic University, Boca Raton, Florida, United States; Florida Atlantic University, Boca Raton, Florida, United States; Florida Atlantic University, Boca Raton, Florida, United States

## Abstract

One quarter of older adults in the U.S. are socially isolated; far more experience subjective loneliness. The potentially negative effects of limited social support and social engagement pose a concern for maintaining the well-being of older individuals, including the effect on cognitive function. To test the hypothesis that cognitive function is associated with social support and social engagement, we employed structural equation modeling (SEM) to analyze data from an ethnically diverse sample (118 African American, 147 Afro-Caribbean, 133 Hispanic, and 235 European American) of 623 community-dwelling older adults age ≥ 65 (173 male, 450 female). Using the model proposed by Cenè et al. (2022), we conceptualized level of social support and engagement as the exposure, emotional state (anxiety, depression, general emotional well-being), and physical function (functional activity, life space, general physical function, and self-rating of health) as mediators and cognitive function as the outcome of interest. Statistically significant direct and indirect effects were found between the three latent variables (emotional state, physical function, and social support and social engagement), indicating that the level of social support and engagement the older individual is related directly and indirectly to cognition (β=-.75, SE=.49, p<.028; β=-0.08, SE=0.795, p=0.017). The SEM models showed goodness of fit with χ2/df=3.001, CFI=0.931, AGFI=0.966, SRMR=0.045, and RMSA=0.058, and explained 35.3% of the variability. These values support the hypothetical model. Results suggest the importance of maintaining existing social support and engagement activities as well as development and testing of approaches to increase social support and engagement in later life.

